# Adjunctive individual meaning-centered psychotherapy plus protocolized fluoxetine for moderate-to-severe adolescent depression

**DOI:** 10.3389/fpsyt.2025.1748005

**Published:** 2026-01-08

**Authors:** Li-Li Sheng, Min Zhang

**Affiliations:** Department of Child and Adolescent Psychiatry, Nanjing Brain Hospital, Nanjing, Jiangsu, China

**Keywords:** adolescents, depression, fluoxetine, individual meaning-centered psychotherapy, PHQ-A

## Abstract

**Objective:**

To determine whether individual, adapted Meaning-Centered Psychotherapy (IMCP), delivered alongside protocolized fluoxetine, improves depressive symptoms and related outcomes in adolescents with moderate-to-severe depression.

**Methods:**

Among 168 adolescents with DSM-5-TR depressive disorder and Patient Health Questionnaire–9 modified for Adolescents (PHQ-A) ≥10, all receiving protocolized fluoxetine, participants were randomized to IMCP+TAU or TAU; the IMCP group completed seven weekly 60-minute sessions. The primary outcome was PHQ-A; secondary outcomes were the Zung Self-Rating Anxiety Scale (SAS), Clinical Global Impressions - Severity and - Improvement (CGI-S/CGI-I), Children’s Global Assessment Scale (CGAS), Rosenberg Self-Esteem Scale (RSES), and Meaning in Life Questionnaire - Presence and Search subscales (MLQ-Presence/Search). Serious adverse events (SAEs) and adverse events (AEs) were recorded throughout the 12-week study period.

**Results:**

Across 12 weeks, the IMCP group showed earlier and larger reductions in depressive symptoms (PHQ-A) than TAU, with advantages evident by week 4 and maintained at weeks 8 and 12; severity distributions shifted more toward milder categories in IMCP. Anxiety (SAS) declined in both groups, with greater improvement in IMCP. Clinician ratings reflected the same pattern: IMCP achieved lower illness severity (CGI-S) and better early improvement (CGI-I), and greater functional gains (CGAS) throughout follow-up. Self-esteem (RSES) rose more in IMCP. Meaning in life-Presence (MLQ-Presence) increased more with IMCP, while Search (MLQ-Search) changed minimally and similarly across groups. Neither group experienced SAEs, and AE frequencies were low and did not differ meaningfully between groups.

**Conclusions:**

Adjunctive IMCP with standardized fluoxetine produced greater 12-week improvements than TAU in symptoms, clinician ratings, functioning, self-esteem, and felt meaning, supporting IMCP as a feasible and effective adjunct.

## Introduction

Adolescence is characterized by rapid biological, cognitive, and neurological changes that reshape psychosocial functioning and relationships, creating a developmental window of heightened vulnerability to mood disturbance (1). Depressive disorder in this period is a major public health concern given its prevalence, early onset, functional impairment across school and relationships, and association with recurrent episodes, suicidality, and long-term psychosocial difficulties (2, 3). Beyond symptomatology, a growing literature links adolescents’ diminished sense of life meaning with higher depressive burden, whereas strengthening meaning and purpose appears to buffer depressive affect and promote well-being (4).

Founded on Viktor Frankl’s teaching regarding the human need for meaning, individual meaning centered psychotherapy (IMCP) is a form of individual psychotherapy initially designed for patients with advanced cancer (5–7). IMCP, is a seven-week program that utilizes a mixture of didactics, discussion and experiential exercises that focus around particular themes related to meaning and advanced cancer, including 1) concepts & sources of meaning:introductions and meaning; 2) cancer & meaning:identity before and after cancer diagnosis; 3) historical sources of meaning: life as legacy that has been given [past] and life as legacy that one lives [present] and gives [future]; 4) attitudinal sources of meaning: encountering life’s limitations; 5) creative sources of meaning: creativity, courage & responsibility; 6) experiential sources of meaning: connecting with life; 7) transitions: reflection & hopes for future (8).

Beyond oncology patients, meaning-centered approaches have been adapted for healthcare providers and caregivers (9–12) and explored in adolescent and young adult (AYA) oncology with promising feasibility and preliminary benefits using in-person and online formats (13). Complementing these findings, school-based “meaning” curricula for youth report improvements in well-being and reductions in depressive and anxious symptoms (4). However, there are currently no rigorously controlled, manualized studies of IMCP specifically for adolescents with primary depressive disorders.

To address this gap, the present study adapts the IMCP curriculum for adolescents with moderate-to-severe depression and evaluates its effectiveness as an adjunct to protocolized fluoxetine. Our primary objective was to determine whether IMCP, delivered in seven weekly sessions alongside standardized medication management, improves depressive symptoms relative to treatment-as-usual (TAU). Secondary objectives are to assess effects on anxiety, clinician-rated severity and improvement, global functioning, self-esteem, and the presence and search for meaning. By embedding a developmentally adapted, manualized IMCP within a medication-standardized trial, this study provides needed evidence on the added clinical value of a meaning-centered approach for adolescent depression.

## Materials and methods

### Ethics statement

The study protocol was approved by the Medical Ethics Committee of Nanjing Brain Hospital and conducted in accordance with the Declaration of Helsinki. Written informed consent was obtained from parents or legal guardians, and written assent from all adolescents.

### Participants

**Figure 1:**
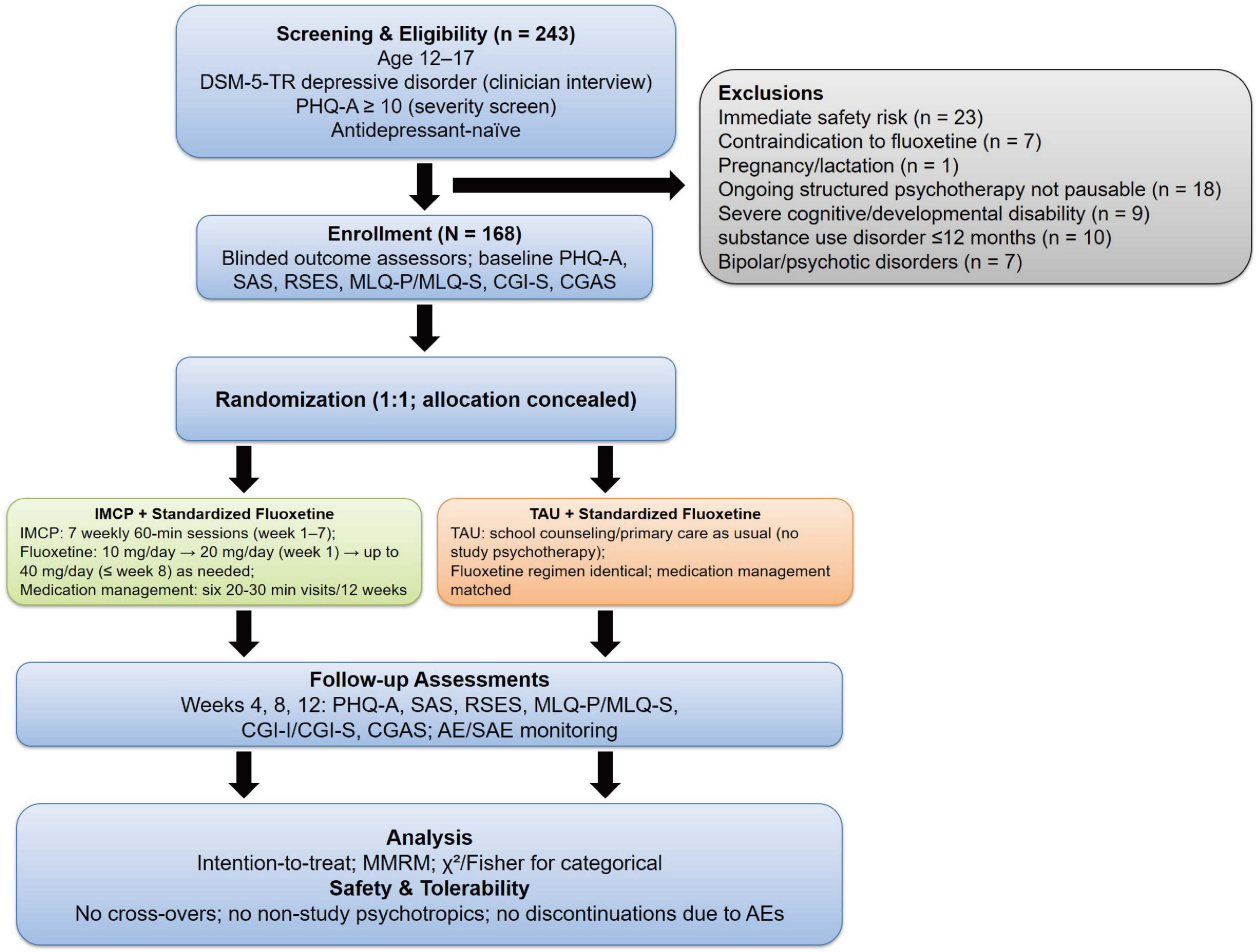
CONSORT study flow and protocol overview. IMCP, Individual Meaning-Centered Psychotherapy; TAU, treatment-as-usual; PHQ-A, Patient Health Questionnaire-9 modified for Adolescents; AE/SAE, adverse/serious adverse event.

Eligible participants were adolescents who met the *Diagnostic and Statistical Manual of Mental Disorders, Fifth Edition, Text Revision (DSM-5-TR)* criteria for a depressive disorder (14) based on a clinician-administered diagnostic interview. Youth aged 12-17 years with Patient Health Questionnaire-9 modified for Adolescents (PHQ-A) ≥ 10 (moderate to severe) were enrolled between January 2024 and January 2025; the PHQ-A was used to index severity for eligibility, not for diagnostic ascertainment. Participants were antidepressant-naïve at consent and all began a standardized fluoxetine regimen (10 mg/day start; 20 mg/day at week 1; up to 40 mg/day by week 8 if needed) with six 20–30-minute medication-management visits over 12 weeks; no non-protocol psychotropic medication or dose changes were permitted. Key exclusions included immediate safety risk (e.g., C-SSRS high-risk ideation/behavior or MADRS item-10 indicating imminent risk) requiring intensive care, medical contraindications to fluoxetine, pregnancy/lactation, current psychological/psychiatric treatment that cannot be discontinued, severe cognitive/developmental disability interfering with participation, substance use disorder within 12 months, bipolar disorder or schizophrenia-spectrum/delusional disorder, inability/unwillingness to complete the program, or any other condition judged inappropriate for participation by the study psychiatrist. Participants must have a stable living situation and a reliable contact for safety follow-up. The CONSORT flowchart was provided in **Figure 1**.

### Sample size calculation and randomization

In a two-group parallel design with two-sided α *=* 0.05 and 85% power, targeting a between-group difference of 3.0 points on PHQ-A (15) and SD *=* 5.6 (16), the required sample size is 63 participants per group. Allowing for 25% attrition, we planned to enroll 84 per group (N = 168). Participants were randomly assigned to one of two groups: (1) IMCP group: a 7-week IMCP program delivered in weekly sessions, or (2) a TAU group. Both groups received the same 12-week protocolized fluoxetine and scheduled medication-management visits; IMCP was delivered only to the intervention arm as an adjunct. A biostatistician generated the allocation sequence using computer-generated permuted blocks of variable sizes, stratified by sex (F/M) and baseline PHQ-A (10–14 *vs.* ≥15). Allocation was concealed via sequentially numbered, opaque, sealed envelopes prepared off-site and opened after baseline assessment. Outcome assessors were blinded to group assignment; participants and therapists were not.

### IMCP program

IMCP program: conducted in an individual format, comprising seven 60-minute sessions over 7 weeks (17), building a shared language of “meaning versus despair versus depression” and progressively activates three primary sources of meaning-creative, experiential, and attitudinal-within a historical/legacy frame. Session 1 established alliance, shared goals, and a brief “meaning-moment” exercise to evoke internal resources; Session 2 revisited core concepts while differentiating self from symptoms to curb rumination and global self-criticism; Session 3 situated identity on a lifeline of legacy, linking past achievements and roles to present and future purpose; Session 4 trained attitudinal choice under constraint and pain, strengthening stance and agency; Session 5 reinstated values through micro-creation, courage, and responsibility via feasible, actionable steps; Session 6 rebuild connection to life through experiential sources-love, beauty/nature, and humor-with practice of savoring and grounding; Session 7 integrated gains in a personal “Meaning Declaration,” and finalizes relapse-prevention and ongoing value-based action plans. The detailed core procedures were summarized in **Table 1**. TAU comprised school counseling and primary-care follow-up on demand, psychoeducation handouts, and usual crisis pathways; structured psychotherapy was not provided by study staff.

**Table 1:** IMCP adolescent depression intervention (12-week)-S1–S7 core procedures.

Session	Objectives	Core procedures
S1	Build working alliance; introduce “meaning–despair–depression”; set shared goals	1) mood thermometer & safety framework 2) brief psychoeducation on the triangle model 3) values card sort 4) goal setting: GAS + functional 5) action plan: smallest actionable step ≤ 2′ + If–Then 6) parent/caregiver 5-min check-in & homework brief
S2	Differentiate self *vs.* symptoms; reduce global self-criticism	1) self-map (school/home/peers/online) 2) ABC three-panel comic → balanced statement 3) three-step self-compassion 4) assign one ABC + balanced statement as homework
S3	Identify past sources of meaning: achievements, roles, turning points	1) 10-cell lifeline 2) evidence cards 3) micro-narrative reauthoring 4) link to one value + one 2-minute action for this week
S4	Choose a stance toward uncontrollable pain/stress	1) trigger inventory (top 3) 2) controllable/uncontrollable two-by-two 3) box breathing ×3 + brief JPMR 4) comparison-stop phrase 5) mid-point check at Week 4: risk screen + progress *vs.* GAS
S5	Link strengths to contribution/action	1) strengths spotting 2) meaning-to-contribution canvas 3) design a micro-project (≤10′/day) 4) consolidate If–Then chain 5) schedule execution across weeks 5–12
S6	Cultivate presence via love/beauty/humor/nature	1) five-sense group-up 2) four-channel practice 3) select “daily 2-minute prescription” 4) align with values 5) add a simple tracker (calm/pleasure 0-10) weeks 6–12

This table summarizes a once-weekly, 12-week meaning-centered program (IMCP), detailing session objectives and core procedures. GAS, Goal Attainment Scaling; JPMR, Jacobson Progressive Muscle Relaxation; ABC, Adversity- Belief -Consequence.

### Outcome measurements

The assessments were administered at baseline, week 4, week 8, and week 12. The 7-week IMCP curriculum concluded at week 7, while standardized fluoxetine management continued through week 12 for both groups; thus, outcome assessments at weeks 8 and 12 captured post-IMCP maintenance under ongoing medication management. The primary outcome was the PHQ-A, a 9-item self-report of depressive symptoms over the prior two weeks scored 0–3 per item (0 *=* Not at all, 3 *=* Nearly every day); items were summed to 0–27 with conventional severity bands 0–4 minimal, 5–9 mild, 10–14 moderate, 15–19 moderately severe, and 20–27 severe (18). Secondary outcomes included: the Zung Self-Rating Anxiety Scale (SAS), a 20-item 4-point scale over the prior two weeks, the raw sum was multiplied by 1.25 to yield a 25–100 standard score (higher = more anxiety) (19); the Meaning in Life Questionnaire (MLQ), comprising two 5-item subscales-Presence (items 1, 4, 5, 6 and item 9 reverse-scored) and Search (items 2, 3, 7, 8, 10)-with items rated 1–7 and summed to subscale totals (higher *=* greater presence/search of meaning) (20); the Rosenberg Self-Esteem Scale (RSES), a 10-item 4-point scale with items 2, 5, 6, 8, 9 reverse-scored and all items summed to a continuous total (range 10-40; higher *=* higher global self-esteem) (21); the Children’s Global Assessment Scale (CGAS), a clinician-rated single score from 1–100 indexing overall functioning (higher *=* better) anchored to the reference week (22); and the Clinical Global Impressions-Severity (CGI-S) and -Improvement (CGI-I), each clinician-rated on 0–7 scales (CGI-S: 1 *=* normal/not at all ill … 7 *=* among the most extremely ill; CGI-I: 1 *=* very much improved … 7 *=* very much worse; 0 *=* not assessed) (23). At each medication-management visit, adverse events (AEs) were proactively elicited using a structured checklist and coded for severity and relatedness; serious adverse events (SAEs) were defined per ICH criteria with immediate reporting. All AEs and SAEs were recorded throughout the 12-week period. Outcome assessors were blinded to group assignment.

### Statistical analysis

All analyses followed an intention-to-treat (ITT) principle. Under an ITT framework, a mixed-effects model for repeated measures (MMRM; fixed effects: group, time, and group×time; random intercept for participant) was used to evaluate longitudinal outcomes and between-group differences at each timepoint via model-based marginal estimates. Residuals and distributional assumptions were examined (Shapiro–Wilk); for prespecified single-timepoint two-group comparisons with non-normality, Mann–Whitney tests were used. Categorical variables used *χ*² or Fisher’s exact tests. Effect sizes were reported as model-based marginal mean differences and standardized mean differences for continuous outcomes, Hodges-Lehmann median differences (and rank-based standardized effects) for non-parametric comparisons, and Cramér’s V (with odds ratios for 2 × 2 tables) for categorical variables. All tests were two-tailed with α = 0.05. All analyses were performed in GraphPad Prism (version 8.0) or SPSS 22.0.

## Results

### Baseline characteristics

Baseline characteristics were well balanced between groups (**Table 2**). Mean age did not differ (IMCP 14.81 ± 1.68 *vs.* TAU 14.65 ± 1.77 years, *P =* 0.582). Sex distribution was similar (female 61.9% *vs.* 58.3%, *P =* 0.753), as was school stage (junior high 41.7% *vs.* 45.2%, *P =* 0.756). Family history of depression showed comparable proportions (29.8% *vs.* 32.1%, *P =* 0.868). Weight categories were also similar across groups (underweight 11.9% *vs.* 9.5%, normal 67.9% *vs.* 73.8%, overweight/obesity 20.2% *vs.* 16.7%; overall *P =* 0.723). These findings indicate no significant baseline imbalances likely to confound treatment effects. Retention was 100% (168/168) with no missing primary or secondary outcomes at weeks 4, 8, and 12. No participant discontinued study treatment, crossed over, or received non-protocol psychotropics.

**Table 2:** Baseline characteristics at enrollment.

Characteristic	IMCP (n = 84)	TAU (n = 84)	Effect sizes	*P* value
Age, years, mean ± SD	14.81 ± 1.68	14.65 ± 1.77	0.09	0.582
Sex			0.04	0.753
Female, n (%)	52 (61.9%)	49 (58.3%)		
Male, n (%)	32 (38.1%)	35 (41.7%)		
School stage			0.04	0.756
Junior high, n (%)	35 (41.7%)	38 (45.2%)		
Senior high, n (%)	49 (58.3%)	46 (54.8%)		
Family history of depression, n (%)	25 (29.8%)	27 (32.1%)	0.03	0.868
Weight category			0.07	0.723
Underweight, n (%)	10 (11.9%)	8 (9.5%)		
Normal, n (%)	57 (67.9%)	62 (73.8%)		
Overweight/obesity, n (%)	17 (20.2%)	14 (16.7%)		

IMCP, Individual Meaning-Centered Psychotherapy; TAU, treatment-as-usual; SD, standard deviation. Effect sizes were reported as Cohen’s d (or Hodges–Lehmann median difference for non-parametric comparisons) for continuous outcomes, and Cramér’s V for categorical variables.

### IMCP produced faster, larger improvements in depressive symptoms and anxiety than TAU in moderate-to-severe adolescent depression

**Figure 2:**
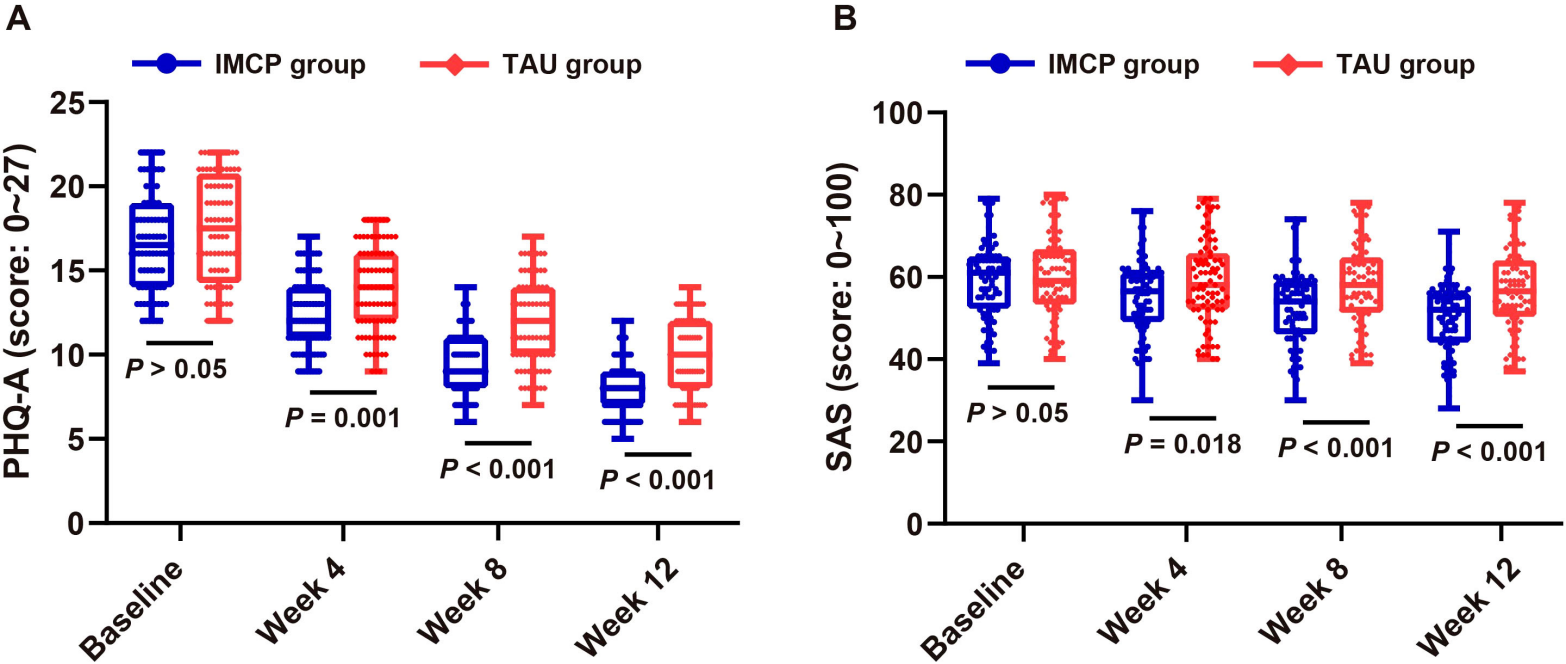
Symptom trajectories in adolescents with moderate-to-severe depression: IMCP *vs.* TAU over 12 weeks. **(A)** Mean PHQ-A scores (higher *=* worse depression) at baseline, week 4, week 8, and week 12 in adolescents with moderate-to-severe depression; **(B)** Mean SAS scores (higher *=* greater anxiety) across the same timepoints; Values are means ± SD (n *=* 84 per group per timepoint). PHQ-A: Patient Health Questionnaire-9 modified for Adolescents; SAS: Zung Self-Rating Anxiety Scale; IMCP: Individual Meaning-Centered Psychotherapy; TAU: treatment-as-usual; SD, standard deviation.

Under an ITT framework, the groups were comparable at baseline on PHQ-A (IMCP 16.76; TAU 17.37; Δ = -0.61, *P* = 0.112). The group × time interaction was significant (*P* < 0.001), indicating a faster decline in depressive symptoms with IMCP versus TAU. Estimated marginal means showed advantages for IMCP from week 4 onward: week 4, 12.55 *vs.* 13.87 (Δ = -1.32, *P* = 0.001); week 8, 9.60 *vs.* 11.92 (Δ = -2.32, *P* < 0.001); week 12, 7.87 *vs.* 10.01 (Δ = -2.14, *P* < 0.001; **Figure 2A**). PHQ-A severity distributions mirrored these findings (**Table 3**): no baseline difference (*P* = 0.214), with a greater shift toward milder symptom categories in IMCP by week 4 (*P* = 0.018) and pronounced advantages at weeks 8 and 12 (both *P* < 0.001). For anxiety, SAS scores (higher = worse) declined in both groups (**Figure 2B**), with faster and larger reductions in IMCP. Baseline was comparable (59.45 *vs.* 59.88; Δ = -0.43, *P* = 0.779). Differences favored IMCP from week 4: -3.96 (*P* = 0.010) at week 4, -5.18 (*P* = 0.001) at week 8, and -6.23 (*P* < 0.001) at week 12, indicating earlier and progressively greater improvement with IMCP.

**Table 3:** PHQ-A severity distribution by group and time (n *=* 84 per group per timepoint).

Timepoint	Severity category (PHQ-A)				Effect size	*P*
	Mild (5-9)	Moderate (10-14)	Moderately severe (15-19)	Severe (≥20)		
Baseline						
IMCP group, n (%)	0 (0.0)	23 (27.4)	43 (51.2)	18 (21.4)	0.10	0.214
TAU group, n (%)	0 (0.0)	21 (25.0)	35 (41.7)	28 (33.3)
Week 4						
IMCP group, n (%)	8 (9.5)	56 (66.7)	20 (23.8)	0 (0.0)	0.19	0.018
TAU group, n (%)	3 (3.6)	45 (53.6)	36 (42.9)	0 (0.0)
Week 8						
IMCP group, n (%)	44 (52.4)	40 (47.6)	0 (0.0)	0 (0.0)	0.52	< 0.001
TAU group, n (%)	17 (20.2)	51 (60.7)	16 (19.0)	0 (0.0)
Week 12						
IMCP group, n (%)	69 (82.1)	15 (17.9)	0 (0.0)	0 (0.0)	0.49	< 0.001
TAU group, n (%)	33 (39.3)	51 (60.7)	0 (0.0)	0 (0.0)

PHQ-A, Patient Health Questionnaire–9 modified for Adolescents; IMCP, Individual Meaning-Centered Psychotherapy; TAU, treatment-as-usual; Effect sizes were reported as Cramér’s V for categorical variables.

### IMCP yielded superior clinician-rated improvement and functional recovery versus TAU in moderate-to-severe adolescent depression

**Figure 3:**
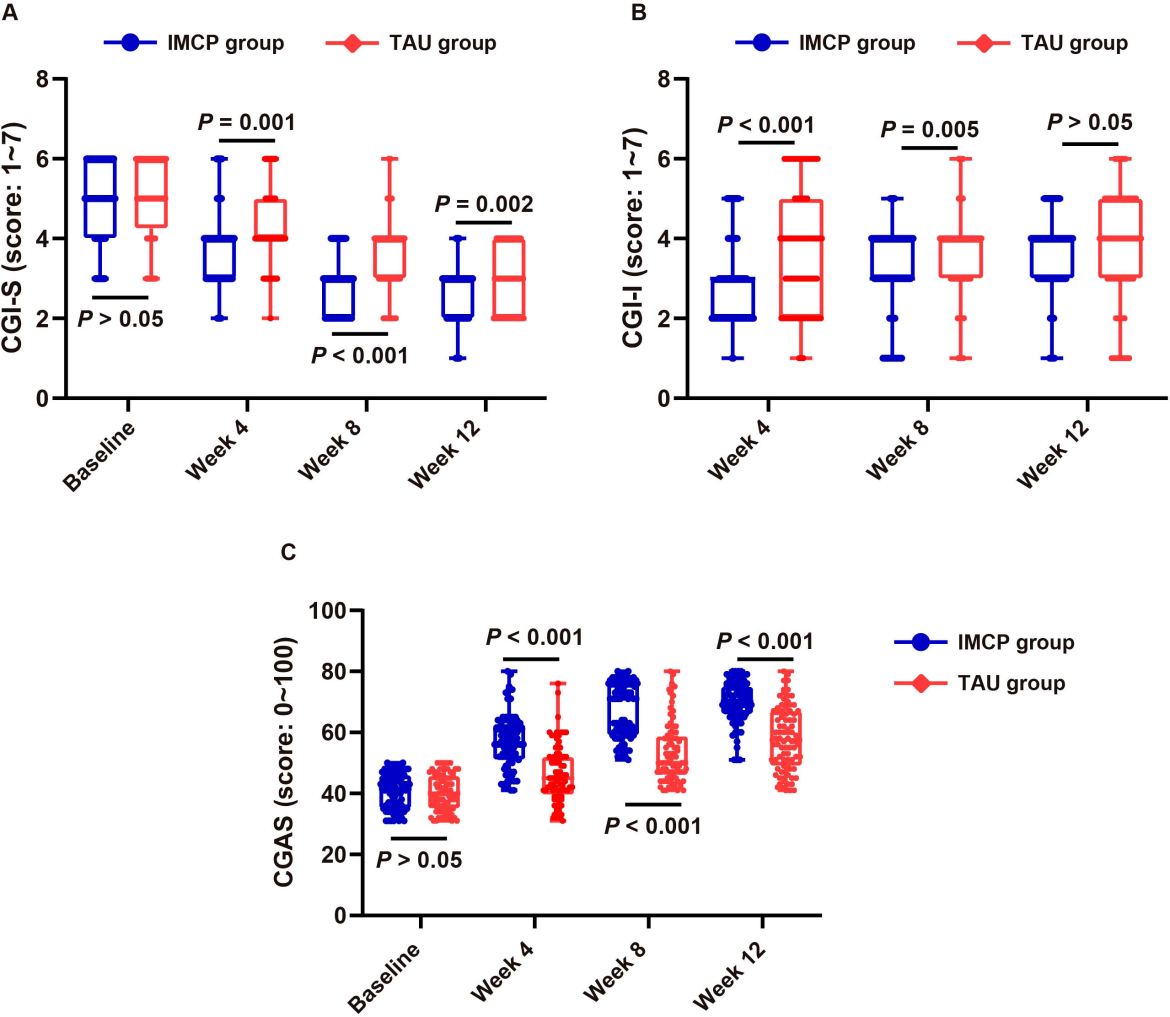
Clinician-rated outcomes in moderate-to-severe adolescent depression: IMCP *vs.* TAU over 12 weeks. **(A)** Mean CGI-S scores (lower = less ill) at baseline, week 4, week 8, and week 12; **(B)** Mean CGI-I scores (lower = greater improvement) at weeks 4, 8, and 12; **(C)** Mean CGAS scores (higher = better functioning) at baseline, week 4, week 8, and week 12. Error bars show SD (n = 84 per group per timepoint). CGI-S, Clinical Global Impressions-Severity; CGI-I, Clinical Global Impressions–Improvement; CGAS, Children’s Global Assessment Scale; IMCP, Individual Meaning-Centered Psychotherapy; TAU, treatment-as-usual; SD, standard deviation.

Under ITT, clinician ratings favored IMCP over TAU. On CGI-S (lower = less ill; **Figure 3A**), the groups were comparable at baseline (4.94 *vs.* 5.01; Δ = -0.07, *P* = 0.650). From week 4 onward, IMCP showed significantly lower severity: Δ = -0.50 (*P* = 0.001) at week 4, Δ = -0.64 (*P* < 0.001) at week 8, and Δ = -0.38 (*P* = 0.002) at week 12. On CGI-I (lower = greater improvement; **Figure 3B**), IMCP achieved better early improvement: week 4 means 2.88 *vs.* 3.71 (Δ = -0.83, *P* < 0.001) and week 8 means 3.06 *vs.* 3.56 (Δ = -0.50, *P* = 0.005), with no difference at week 12 (3.60 *vs.* 3.67; Δ = -0.07, *P* = 0.714). On CGAS (higher = better functioning; **Figure 3C**), baseline was similar (40.73 *vs.* 40.29; Δ = 0.44, *P* = 0.626), while IMCP yielded larger gains at all follow-ups: +10.62 (*P* < 0.001) at week 4, + 13.81 (*P* < 0.001) at week 8, and +11.95 (*P* < 0.001) at week 12. These findings indicate that IMCP accelerated functional recovery and clinician-rated improvement beyond standardized medication management alone.

### IMCP improved self-esteem versus TAU in moderate-to-severe adolescent depression

**Figure 4:**
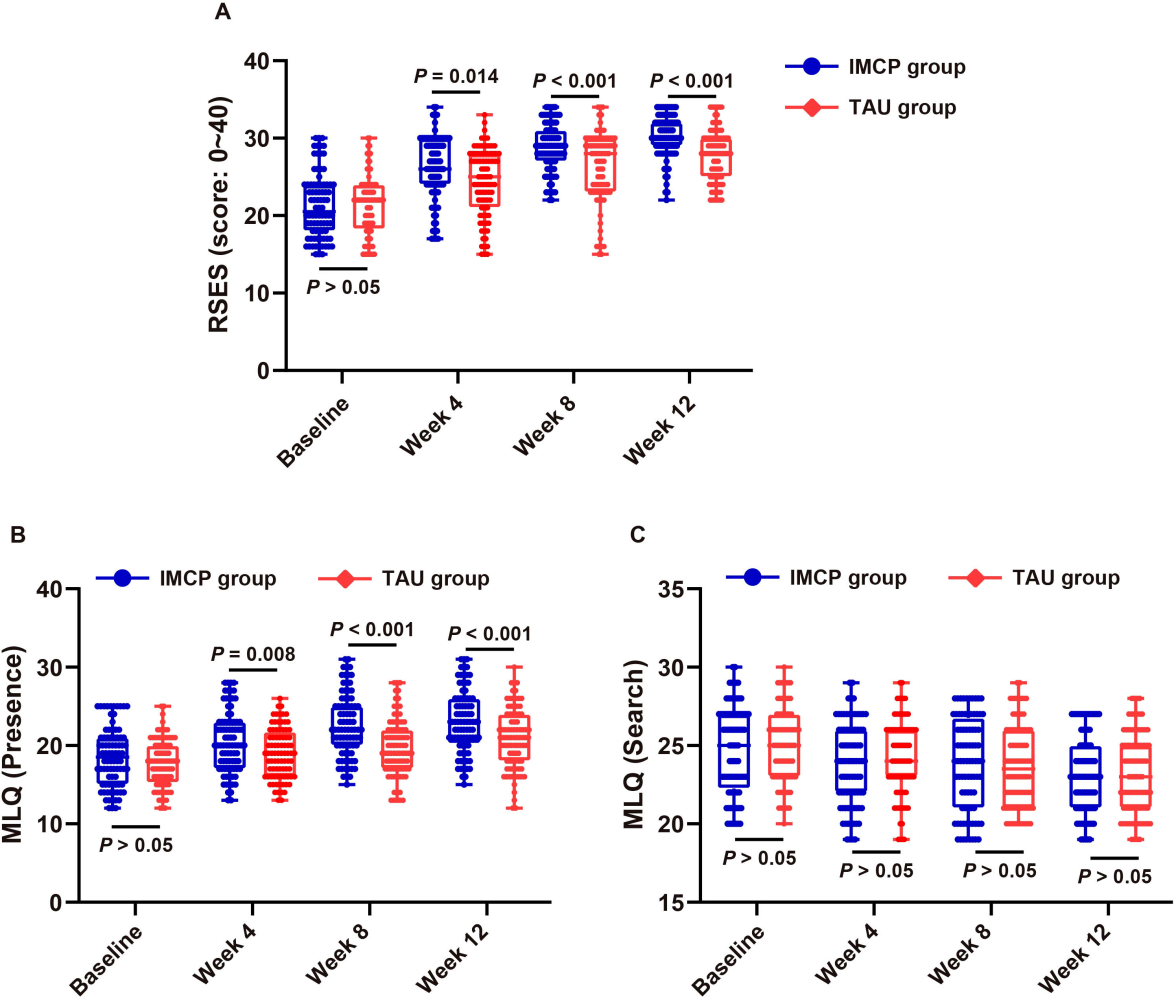
Psychosocial outcomes in moderate-to-severe adolescent depression: IMCP *vs.* TAU over 12 weeks. **(A)** Mean RSES scores (higher = greater self-esteem); **(B)** Mean MLQ–Presence scores (higher = more felt meaning); **(C)** Mean MLQ-Search scores (higher = more striving for meaning) at baseline, week 4, week 8, and week 12. Error bars show SD (n = 84 per group per timepoint). RSES, Rosenberg Self-Esteem Scale; MLQ, Meaning in Life Questionnaire (Presence, Search subscales); IMCP, Individual Meaning-Centered Psychotherapy; TAU, treatment-as-usual; SD, standard deviation.

Under ITT, self-esteem (RSES; higher = better; **Figure 4A**) increased over time in both groups, with larger gains in IMCP. Baseline scores were comparable (21.31 *vs.* 21.36; *P* > 0.05). IMCP exceeded TAU at week 4 (26.05 *vs.* 24.27; *P* = 0.014), with advantages widening at week 8 (28.81 *vs.* 26.27; *P* < 0.001) and maintained at week 12 (30.17 *vs.* 27.90; *P* < 0.001), indicating earlier and greater improvement versus TAU.

### IMCP enhanced the felt presence of meaning with stable search of meaning versus TAU in moderate-to-severe adolescent depression

Under ITT, MLQ-Presence (higher = more felt meaning; **Figure 4B**) increased in both groups, with larger gains in IMCP. Baseline was comparable (18.52 *vs.* 17.82; Δ = +0.70, *P* = 0.191). Between-group differences favored IMCP at week 4 (+1.48, *P* = 0.008), week 8 (+2.80, *P* < 0.001), and week 12 (+2.23, *P* < 0.001). In contrast, MLQ-Search (higher = more striving for meaning; **Figure 4C**) showed a small decline in both groups with no between-group differences at any time (all *P* ≥ 0.05): baseline Δ = -0.25 (*P* = 0.542); week 4 Δ = -0.40 (*P* = 0.310); week 8 Δ = +0.12 (*P* = 0.783); week 12 Δ = -0.06 (*P* = 0.879).

### Comparable adverse events over 12 weeks between the IMCP and TAU groups

No SAEs occurred in either group, no participant discontinued study treatment due to AEs, and there were no cross-overs or non-protocol psychotropic medications. As shown above and in **Table 4**, AE rates were infrequent and similar between groups, with no meaningful between-group differences (all *P* > 0.05). Gastrointestinal discomfort (e.g., nausea, abdominal discomfort, diarrhea, dry mouth) occurred in 7/84 (8.3%) in IMCP vs 9/84 (10.7%) in TAU (*P* = 0.794); central nervous system/sleep-related symptoms (e.g., headache, insomnia or hypersomnia, fatigue, vivid dreams) in 8/84 (9.5%) vs 10/84 (11.9%; *P* = 0.804); appetite/weight change (e.g., reduced appetite, mild weight fluctuation) in 3/84 (3.6%) vs 4/84 (4.8%; *P* = 1.000); and activation-like symptoms (e.g., restlessness, irritability, transient anxiety uptick) in 2/84 (2.4%) vs 1/84 (1.2%; *P* = 1.000).

**Table 4:** Adverse events over 12 weeks.

AE category	IMCP (n=84) n (%)	TAU (n=84) n (%)	*P*	Effect size
Gastrointestinal discomfort	7 (8.3%)	9 (10.7%)	0.794	0.041
Central nervous system/sleep-related	8 (9.5%)	10 (11.9%)	0.804	0.038
Appetite/weight change	3 (3.6%)	4 (4.8%)	1.000	0.030
“Activation”-like symptoms	2 (2.4%)	1 (1.2%)	1.000	0.045

IMCP, Individual Meaning-Centered Psychotherapy; TAU, treatment-as-usual; Effect sizes were reported as Cramér’s V for categorical variables.

## Discussion

This randomized trial demonstrated that, in adolescents with moderate-to-severe depression, adding an adapted IMCP to standardized fluoxetine yields faster and larger improvements over 12 weeks than TAU across depressive symptoms, anxiety, clinician ratings, functioning, self-esteem, and the presence of meaning.

These results extend the MCP evidence base - originally established in adults with cancer in group and individual formats (5–8) - to adolescents with primary depressive disorders. Converging youth data further support a meaning-centered approach: a randomized clinical trial of virtual logotherapy improved health-promoting lifestyle among single-parent adolescent girls (24); an eight-module online mindfulness-based logotherapy pilot reduced depressive symptoms in cyberbullied adolescents (25); and expert commentary has argued for systematic integration of existential (logotherapy) principles into personalized adolescent depression care (26). Conceptually, MCP and logotherapy share a common Franklian foundation—freedom of will, will to meaning, and creative/experiential/attitudinal sources of meaning—with MCP essentially operationalizing and manualizing these principles for clinical delivery (27). Related literatures in adolescents and young adults with cancer show feasibility and signals of benefit for meaning-oriented programs delivered in person or online (13), and school-based “meaning” curricula report gains in well-being with reduced depressive/anxious symptoms, albeit largely from quasi-experimental designs (4). Building on these strands, our trial advances the field by testing a developmentally adapted IMCP while holding pharmacotherapy constant via a protocolized fluoxetine pathway, and by demonstrating convergent advantages across patient-reported, clinician-rated, and functional endpoints.

We hypothesize that the multi-domain gains arose from a sequenced pathway beginning with value clarification, proceeding to concrete goals, continuing with enactment, reinforced by experiential practices, and consolidated over time. First, the values card sort likely increased coherence and well-being in a manner consistent with school-based value/self-affirmation research, which has demonstrated small-to-moderate benefits - helping to account for increases in MLQ-Presence and self-esteem (28). Next, GAS goal setting paired with If–Then implementation intentions turns abstract priorities into small, executable steps; adolescent cluster-randomized work that embeds structured planning shows shifts in health-related determinants and behaviors at scale, aligning with our early gains on CGI and CGAS (29). The ABC three-panel exercise combined with three-step self-compassion likely reduced rumination and global self-blame, consistent with meta-analytic evidence that higher self-compassion in youth is robustly associated with lower depression and anxiety (30). In S4, brief bouts of paced/box breathing and progressive muscle relaxation down-regulate physiological arousal and stabilize affect; recent trials and meta-analyses show that breathwork and PMR reduce stress, anxiety, and depressive symptoms—including in adolescents—supporting our SAS and PHQ-A trajectories (31). Strengths spotting together with micro-contribution projects converts capability and a sense of “mattering” into daily prosocial action, echoing strength-based adolescent interventions that improve resilience, self-efficacy, and other positive mental-health indicators—plausible drivers of our CGAS, RSES, and MLQ-Presence gains (32). Finally, the experiential channel work in S6 (love, beauty/nature, humor) targets an experiential source of meaning emphasized in youth meaning-centered programs and reviews, which helps explain a larger rise in Presence with relative stability of Search (4).

Limitations include: (1) Participants/therapists were not blinded to IMCP, although assessors were blinded. (2) Follow-up was 12 weeks; durability/relapse prevention remain unknown. (3) Outcomes relied primarily on validated self-reports plus clinician ratings without uniform objective corroboration (e.g., attendance/grades). (4) Retention was 100% with complete outcome ascertainment—an advantage for internal validity but atypical in adolescent depression trials; this may limit generalizability and likely reflects short follow-up, single-site setting, proactive retention procedures, and enrollment of more adherent families. (5) Single-site design and restriction to antidepressant-naïve, moderate-to-severe cases under a standardized fluoxetine protocol limit generalizability to other severities, comorbidities, settings, or medications. (6) Although safety was closely monitored and no discontinuations occurred due to AEs, the study was not powered for rare SAEs. (7) Enrollment occurred after peak pandemic restrictions, but pandemic-related system changes—especially expanded telemedicine—could act as time-related confounders; prior work describes the “double-edged” effects of telemedicine on clinical workflows and psychosocial outcomes (33). We did not stratify by care modality; future multi-site studies with longer follow-up should log modality (in-person vs telemedicine), include objective functional measures, and test robustness under routine levels of attrition.

## Conclusions

Adjunctive IMCP, delivered alongside standardized fluoxetine, produced faster and larger 12-week improvements than TAU in adolescent depression across symptoms, clinician ratings, functioning, self-esteem, and the felt presence of meaning. These findings support IMCP as a feasible, theory-consistent adjunct to medication management for adolescents with moderate-to-severe depression.

## Data Availability

The raw data supporting the conclusions of this article will be made available by the authors, without undue reservation.
